# An Exploration of the Effect of Hemodynamic Changes Due to Normal Aging on the fNIRS Response to Semantic Processing of Words

**DOI:** 10.3389/fneur.2014.00249

**Published:** 2014-12-01

**Authors:** Mahnoush Amiri, Philippe Pouliot, Clément Bonnéry, Paul-Olivier Leclerc, Michèle Desjardins, Frédéric Lesage, Yves Joanette

**Affiliations:** ^1^Laboratory of Optical and Molecular Imaging, Biomedical Engineering, Polytechnique Montreal, Montreal, QC, Canada; ^2^Laboratory of Neuropsychology of Language, Research Center; Aging Neuroscience, Institut Universitaire de Gériatrie de Montréal, Montreal, QC, Canada; ^3^Montreal Heart Institute, Montreal, QC, Canada; ^4^Biomedical Engineering Institute, University of Montreal, Montreal, QC, Canada; ^5^Faculty of Medicine, University of Montreal, Montreal, QC, Canada

**Keywords:** cognitive aging, language, semantics, functional NIRS, anatomical MRI, baseline physiology, hemodynamic response, ASL

## Abstract

Like other neuroimaging techniques assessing cerebral blood oxygenation, near-infrared spectroscopy (NIRS) has been applied in many neurocognitive studies. With NIRS, neural activation can be explored indirectly via hemodynamic changes in the imaged region. In studies of aging, changes in baseline physiology and brain anatomy confound NIRS measures seeking to investigate age-related changes in neuronal activity. The field is thus hampered by the complexity of the aging process itself, and statistical inferences from functional data acquired by optical imaging techniques must be interpreted with care. Multimodal integration of NIRS with both structural and baseline physiological assessments is crucial to avoid misinterpreting neuroimaging signals. In this study, a combination of two different optical techniques, anatomical MRI and Arterial Spin Labeling (ASL), was used to investigate age-related changes in activation during a lexical-semantic processing task. Quantitative analysis revealed decreased baseline oxyhemoglobin and cerebral blood flow in the older adults. Using baseline physiology measures as regressors in the investigation of functional concentration changes when doing analyses of variance, we found significant changes in task-induced areas of activity. In the right hemisphere, more significant age-related activity was observed around the junction of the inferior frontal gyrus and inferior precentral sulcus, along with engagement of Wernicke’s area. In the left hemisphere, the degree and extent of frontal activation, including the dorsolateral prefrontal cortex and inferior frontal gyrus, differed between age groups. Measuring background physiological differences and using their values as regressors in statistical analyses allowed a more appropriate, age-corrected understanding of the functional differentiations between age groups. Age-corrected baselines are thus essential to investigate which components of the NIRS signal are altered by aging.

## Introduction

Given the growing proportion of elderly adults in the population due to increased longevity, studies investigating and promoting healthy cognitive aging are of the utmost importance. By 2050, the number of elderly individuals will be 16% higher than the number of children and adolescents under 15 years (1). In this context, the number of dementia cases in the aging population is expected to grow exponentially. Prevalence studies in all world regions estimate that 24.3 million people currently have dementia and predict that the number of persons with Alzheimer’s disease will double every 20 years, rising to 81.1 million by 2040 (2). This trend supports the importance of characterizing the mechanisms underlying healthy cognitive aging in order to optimize healthy aging and possibly contribute to delaying the manifestations of dementia.

Normal aging is characterized by significant modifications of the brain’s anatomy and physiology, which vary depending on the brain region and component (3). The overall volume and weight of the brain decrease with each decade of age but displays regional variability. For example, in a five-year longitudinal study, Raz and colleagues have examined age-related differences in regional brain volume (4). A significant negative correlation between age and volume of the lateral prefrontal cortex, orbitofrontal cortex, and prefrontal white matter was observed. In the temporal association cortices, a more moderate shrinkage with age was also found. These anatomical changes are associated with widening sulci and synaptic loss, but negligible neural loss has been observed.

Age also affects sensory and cognitive abilities but in a heterogeneous fashion, varying with cognitive domain. A first hypothesis to explain this observation is that there is a correlation between structural changes and functions. Depending on the cognitive domain (5) and individual characteristics (6), cognitive abilities are affected differently. Among brain functions that are better preserved with aging, older adults have shown a good preservation of semantic word processing and conceptual knowledge organized to depict the relationship between words and stored knowledge of the world. Given that the language-related brain regions (e.g., prefrontal and superior temporal cortices) (7–10) are affected by age (11, 12), investigating the mechanisms underlying the relative preservation of language abilities is essential to better understand how the aging brain handles structural and physiological decline.

A major obstacle to these studies is that interindividual variability in cognitive domains increases with aging and this makes it difficult to apply inferences from individual observations over the entire elderly population. To interpret this variability, one can posit that interindividual differences are mainly due to large variations in the anatomical and/or neurophysiological structures underpinning cognitive performance with age (13). An alternative hypothesis is that some older adults compensate for cognitive aging by either adapting compensatory processing procedures by means of an inter- and/or intrahemispheric functional reorganization or changing cognitive strategies (5, 6, 14, 15), relying on what has been conceptualized as their cognitive reserve (16). With the aim of revealing cognitive changes associated with age, numerous neuroimaging studies have investigated age-related neurophysiological changes associated with functional brain activities (17–20). Despite overall similarities in basic neuronal activity in young and older adults, older individuals show less activity in some brain regions and/or over-recruitment of other brain regions (21) in response to complex tasks. Over-recruitment can be interpreted as a compensatory mechanism or as an indication of neuronal inefficiency. Thus, the challenge in cognitive aging research is to distinguish between these two mechanisms.

To investigate the complex phenomenon of aging, functional near-infrared spectroscopy (fNIRS) has been used in cognitive neuroscience because of its moderate running costs, portability and potential for examinations in a natural setting (22). This non-invasive imaging technique allows researchers to probe the hemodynamic response evoked by neural activity in the first centimeters of cortical tissues. By emitting near-infrared light (650–950 nm) through the scalp and measuring the photons attenuated by absorbing compounds primarily composed of oxy- and deoxygenated hemoglobins (HbO_2_ and HbR, respectively), estimates of neural activation can be recovered (23, 24). One advantage of fNIRS measures over the blood-oxygen level dependent (BOLD) signal obtained from functional magnetic resonance imaging (fMRI) is its ability to measure oxygenation level. However, in hemodynamic-based functional neuroimaging techniques, such as fMRI and fNIRS, neural activity is measured indirectly through neurovascular coupling as a function of changes in cerebral blood flow (CBF), blood volume, and oxygenation (25). Signals are therefore subject to interpretation difficulties due to the ambiguous interaction of the neurophysiology and vasculature underpinning the hemodynamic response (26–29). Thus, changes in measured activation response are related not only to neuronal activity but also to modifications of the underlying physiology with age. There is evidence from the literature that global CBF decreases with age, while the cerebral metabolic rate of oxygenation (CMRO_2_) increases (13, 19, 30), and that microvascular capacity in response to strong demand for oxygenation also declines (17). It is therefore essential for studies to consider these confounding factors if they aim to distinguish the observed physiological changes with age from the underlying neuronal activation in response to a cognitive stimulus.

Other methodological difficulties specific to the NIRS signal are partial volume effects (24) and tissue optical properties that change with age (31, 32). The age-related changes in tissue properties and capillary circulation in the skin (33) and how these changes interact with light propagation in the head may bias NIRS measurements when young and elderly individuals are compared. Time-resolved spectroscopy (TRS) systems provide measure of optical properties of cerebral tissues with the ability to distinguish between superficial layers [skin, skull, and cerebrospinal fluid (CSF)] and brain tissue. Thus, intra- and extracerebral hemoglobin concentrations can be determined for each individual separately (34).

The aim of the present study was to assess the physiological and functional changes that occur in parts of the language processing network during normal aging by means of a lexical-semantic decision task and two imaging techniques: anatomical and blood perfusion (arterial spin labeling; ASL) MRI and fNIRS, as well as time-domain optical imaging TRS. By integrating each individual’s baseline CBF, oxy- and deoxyhemoglobin concentrations, and structural characteristics, obtained with ASL-MRI, TRS, and anatomical MRI, respectively, with the functional hemodynamic responses from NIRS, the goal of this study was to investigate the effect of intrinsic interindividual variability on the hemodynamic responses measured. Specifically, we hypothesized that each individual’s baseline physiology, reflecting his or her neurovascular health, was related to the preservation of semantic memory and cognitive performance. We also hypothesized that higher levels of CBF and oxygen saturation (SatO_2_) from TRS measurements should account for the percentage changes of [HbO_2_] and [HbR] in response to our lexical-semantic decision task. Controlling for these age-related factors is crucial if one wishes to distinguish the presumed age-related neurofunctional reorganization of the brain for cognitive ability, such as the semantic processing of words, from the basic neurophysiological changes linked to the aging brain’s hemodynamics.

## Materials and Methods

### Participants and protocol

In this study, 46 healthy French-speaking individuals divided into two groups of elderly people (*n* = 23), aged 65–75 (mean age = 69.6 ± 4.1), and young people (*n* = 23), aged 20–35 (mean age = 23.4 ± 2.7), were recruited. Because language knowledge is embedded in the social and cultural context, we restricted our participants to French speakers from Quebec. The elderly cohort was chosen from this specific age bracket because of the delicate transition to old age ( >65) and the increased prevalence of cognitive decline (from 4.97 to 24.19%) after the age of 80 (35). The study was approved by the ethics committee of the Institut universitaire de gériatrie de Montréal (IUGM) and all participants gave their written consent. Exclusion criteria were claustrophobia, hypertension or any cardiovascular disease, smoking, thyroid dysfunction, diabetes, taking any medication known to be vasoactive, as well as psychiatric or neurological illness. Participants were all right-handed according to the Edinburgh Handedness Inventory (36, 37). For the measurement of baseline cerebral blood perfusion, they were also asked to abstain from drinking coffee the day of acquisition (38, 39).

Participants were screened for their level of cognitive performance by standardized cognitive assessments including the Trail Making Test A/B (40), the Montreal Cognitive Assessment (MoCA; (41), and five subtests of the short-form Wechsler Adult Intelligence Scale (WAIS-III; (42–44), namely Vocabulary, Block Design, Similarities, Matrix Reasoning, and Direct and Inverse Digit Spans. In this way, it was possible to exclude those with mild cognitive decline according to age-corrected norms. These tasks assess phonological short-term memory storage as well as processing capacities and evaluate general intellectual ability, planning, visual exploration, attention, mental flexibility, and verbal inhibition.

The activation task represents a robust, well-studied lexical-semantic task: lexical decision (45). Stimuli were chosen from the specific categories of non-action words (nouns) denoting non-living objects in order to isolate the peripheral effects in networks associated with semantic processing. Words generated from a French lexical database (OMNILEX database from the Cognitive Psychology of Language Laboratory, University of Ottawa, Canada) were matched according to their lexical frequency, grammatical category (nouns), age of acquisition, orthographic structure, and length in letters. It is important to note that, in visual lexical decision tasks, word length affects reaction time (RT), with stable RTs for words four to six letters long (46). Concreteness of the words (abstract vs. concrete) was manipulated by the imageability index on a scale of 1–7 to investigate the effect of word imageability within a lexical decision test (*n* = 60 for each category). Pseudo-words were then created from the real words (*n* = 120) by changing two consonants. All items (words and pseudo-words) were then matched by bigram frequency and length in letters (Lexique database, Paris Descartes University, France). A pilot study of 15 young adults was done prior to the main study to eliminate outliers within each category. Participants were presented with words and pseudo-words on the screen and were instructed to answer whether or not the letter string constituted a real word. Each trial started with a fixation point (+) that appeared at the center of the screen and was followed by the stimulus. A blank screen provided time to answer with a yes/no button on the computer keyboard. The task was executed using E-prime software (version 2.1), which also recorded the RTs and correct responses.

**Figure 1:**
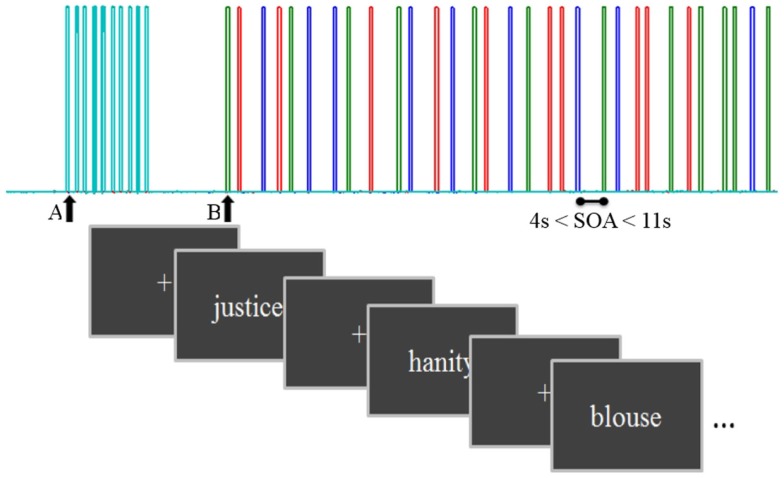
**A schema of the task diagram with inter stimulus interval = 1.36 s and stimulus onset asynchrony from 4 to 11 s**. Triggers from the computer presenting the task were sent to the NIRS computer after synchronization: A, start control task and B, start main task. Each color bar represents a different condition: concrete, abstract, and pseudo-words.

The paradigm was designed in an event-related (ER) fashion. The ER design presented each stimulus at a specific time, allowing the investigation of the evoked hemodynamic response delayed by 2–3 s from stimulus-induced neuronal activity (47). Stimuli from different categories (e.g., experimental conditions; word vs. pseudo-word.) were presented in a random intermixed order for 4–11 s (Figure 1).

### Diffuse optical measurements

#### Near-infrared spectroscopy

**Figure 2:**
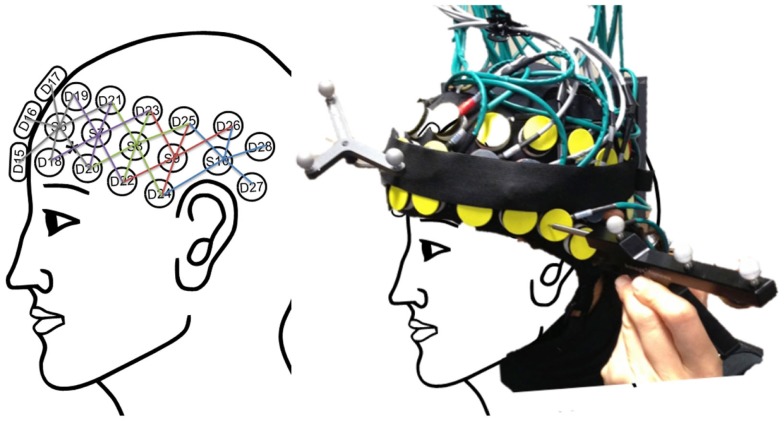
**Left: the schema of the multi-distance optical source-detector design based on the 10–20 standard to cover brain-language regions**. Right: the home-made optical helmet used in this study with the reference and pointer of the stereotaxic system used to register optode positioning for later coregistration on each participant’s anatomical image.

Task-induced changes in optical intensity were measured with a 32-channel continuous wave NIRS instrument (TechEn CW6) with a sampling rate of 25 Hz. TechEn uses two continuous frequency modulated wavelengths at 830 and 690 nm, within a wavelength range where HbO_2_ and HbR are the dominant light absorbers. Using two different wavelengths, we were able to assess the changes in hemoglobin concentration from absorption coefficients (μ_a_) by measuring light attenuation (modified Beer–Lambert law; (23). In this study, we used two patches of 29 channels (a combination of 5 sources and 14 detectors) for each hemisphere, covering the entire frontal and temporal regions of the cortex (Figure 2). To reliably position the posterior edge of the optical helmet, we used the electrode positions Fp_0_ and inion as references according to the 10–20 EEG system.

#### Time-resolved spectroscopy

A TRS system using four pulsed lasers with wavelengths of 690, 750, 800, and 850 nm, temporally multiplexed, illuminated the participant’s forehead. Four detectors at distances of 10, 15, 25, and 30 mm from the point of illumination collected the backscattered light and were then focused on the detection surface of photon-counting avalanche photodiodes with a 20× microscope objective. The experiment took place in a dark room to reduce the noise on the single-photon counter detectors.

The measurements obtained were fit to a double-layer analytical model (34). Thus, the head was modeled as a heterogeneous medium, with the first layer consisting of skin, skull, and CSF and the second layer including both gray and white matter. The model yielded absolute estimates of the optical properties [absorption (μ_a_) and scattering (μ_s_) coefficients] in each layer.

### MRI acquisition

Anatomical MR images were obtained on a 3T Siemens Trio MRI (Siemens Medical Solutions, Erlangen, Germany) using a 32-channel receive-only head coil at the Unité de Neuroimagerie Fonctionnelle of the IUGM. A volumetric Magnetization Prepared Rapid Gradient Echo (MPRAGE) sequence was used to acquire a high-resolution T1-weighted 3D anatomical image, using the following parameters: TR = 2.3 s, TE = 2.91 ms, TI = 900 ms, flip angle = 9°, FOV = 240 × 256, voxel size = 1 × 1 × 1 mm^3^. This sequence was followed by an ASL sequence at rest, without sensory deprivation. The imaging sequence was a PICORE labeling geometry (20) and Q2TIPS tag duration control (48) to quantify the baseline CBF (CBF_0_; (49). A post-label delay of 900 ms and label duration of 1500 ms were used, with repetition time (TR) and echo time (TE) of 3 s and 20 ms, respectively. The ASL signal is evoked by the local magnetization differences following the diffusion of the magnetically labeled blood to quantitatively measure blood perfusion. A single M_0_ scan was also acquired to compute the blood perfusion parameters. This acquisition was done with the same parameters as the ASL sequence except for the TR, which was set to be very long (10 s) to yield a measurement of the fully relaxed magnetization. The whole acquisition including the MPRAGE took approximately 20 min.

### Coregistration

A stereotaxic system (Brainsight, Rogue Research Inc.) was used to align anatomical images from each individual’s MRI and the patch holding the optical fibers. The registered positions of each optode were then mapped into normalized brain coordinates from Montreal Neurological Institute (MNI) template for group analysis.

## Data Analysis

### Behavioral and task performance

*Z*-scores for each cognitive test were calculated from the normative reference data available for different age groups. Only participants whose results were above the normal guideline were kept for further analysis. The RTs and the accuracy of responses to the lexical-semantic task were analyzed using SPSS (IBM, New York, USA). A two-way repeated measures analysis of variance (ANOVA) was applied to RTs for correct answers as a dependent variable with the factors of age (young, elderly) and condition (word, pseudo-word).

### NIRS data signal processing and statistical analysis

Both signal processing (heart rate regression, intensity to concentration conversion, normalization, and smoothing) and statistical analysis (general linear model, GLM) were performed using an SPM8-compatible toolbox made in-house (50) based on NIRS-SPM v3.2; NIRS10.

Changes in optical density, ΔOD, were computed from emitted and received photon fluence Φ:

$$\Delta \mathrm{OD}\left(t,\lambda \right)=-\ln\left(\frac{\Phi \left(t,\lambda \right)}{\Phi_{0}\left(t,\lambda \right)}\right)$$

A heart rate analysis was done to eliminate channels without physiological signals. A coregistration of the source-detector positions on the MNI’s MRI template was done to ensure coherent optode positioning for group analysis. Optical signals were then transformed into hemoglobin concentrations *C*_HbO2_ and *C*_HbR_, applying the modified Beer–Lambert law with

$$\left[\begin{array}{c} C_{\text{HbO2}}\text{(}t\text{)}\\ C_{\text{HbR}}\text{(}t\text{)} \end{array}\right]={\left[\begin{array}{cc} {\text{ε}}_{\text{HbO2}}^{\lambda_{1}} & {\text{ε}}_{\text{HbO2}}^{\lambda_{2}}\\ {\text{ε}}_{\text{HbR}}^{\lambda_{1}} & {\text{ε}}_{\text{HbR}}^{\lambda_{2}} \end{array}\right]}^{1}\left[\begin{array}{c} \Delta \text{OD(}t,\lambda_{1}\text{)/}(d\text{.}\ell_{\text{DPF}}(\lambda_{1}))\\ \Delta \text{OD(}t,\lambda_{2}\text{)/}d\text{.}\ell_{\text{DPF}}(\lambda_{2})) \end{array}\right]$$

where λ_DPF_ is the differential path-length factor accounting for the random photon trajectory between each source and detector (32).

Hemoglobin concentration changes were filtered with a Gaussian kernel (1.5 s FWHM) and high pass filtered by a second-order Butterworth filter with a cutoff frequency of 0.01 Hz. The significance of each effect of interest (abstract, concrete, and pseudo-word) was determined using the theory of Gaussian fields (47). A GLM was fit using a canonical hemodynamic response function (HRF). Contrasts over sessions (intrasubject) were analyzed using a fixed effects model, while testing for contrasts in the intersubject analysis was done by estimating the ratio of the random effects variance to the fixed effects variance. An expected Euler correction based on Lipschitz–Killing curvatures was applied to the threshold on the HbR/HbO_2_ *t*-statistic images to account for the spatial correlation. The GLM method was based on the precoloring method of NIRS-SPM toolbox (51) for noise treatment.

### TRS data

To determine the value of the background absorption and scattering coefficients of the brain, a reflectance curve was fit for each source-detector pair of the time-domain system (34). The curve-fitting procedure was done by a non-linear optimization MATLAB function (*Isqcurvefit*) with fit parameters of absorption and reduced scattering coefficient (μ_a_ and μ_s_, respectively) and amplitude to the theoretical temporal point spread function (TPSF). We applied the appropriate analytical model to fit the reflectance curve. This model was validated by applying a Monte Carlo simulation and with *a priori* information about the thickness of the first layer, including skin, skull, and CSF, obtained from the segmented anatomical MR images (using SPM8). With a high-resolution T1-weighted anatomical image, the maximum errors on the hemoglobin concentrations were expected to be no more than 15% (34).

To determine hemoglobin concentrations from optical parameters, we assumed that oxy- and deoxyhemoglobin and water were the dominant absorbers between the 690 and 850 nm wavelengths. The linear system describing the relationship between the extinction coefficient ε(λ) (taken from the literature) and the absorption coefficient μ_a_(λ) (calculated from TRS measures) is given by

$$\begin{array}{llll} \mu_{a}\left(\lambda \right) & =2.303\cdot \varepsilon \left(\lambda \right)\cdot C\; & & \;\\ \left[\begin{array}{c} {\mu \;\;}_{a}\left(\lambda_{1}\right)\\ {\;\;\mu \;\;}_{a}\left(\lambda_{2}\right)\\ {\;\;\mu \;\;}_{a}\left(\lambda_{3}\right)\\ {\;\;\mu \;\;}_{a}\left(\lambda_{4}\right) \end{array}\right] & =\left[\begin{array}{ccc} \varepsilon_{\mathrm{Hb}O_{2}}\left(\lambda_{1}\right) & \varepsilon_{\mathrm{HbR}}\left(\lambda_{1}\right) & \varepsilon_{H_{2}O}\left(\lambda_{1}\right)\\ \varepsilon_{\mathrm{Hb}O_{2}}\left(\lambda_{2}\right) & \varepsilon_{\mathrm{HbR}}\left(\lambda_{2}\right) & \varepsilon_{H_{2}O}\left(\lambda_{2}\right)\\ \varepsilon_{\mathrm{Hb}O_{2}}\left(\lambda_{3}\right) & \varepsilon_{\mathrm{HbR}}\left(\lambda_{3}\right) & \varepsilon_{H_{2}O}\left(\lambda_{3}\right)\\ \varepsilon_{\mathrm{Hb}O_{2}}\left(\lambda_{4}\right) & \varepsilon_{\mathrm{HbR}}\left(\lambda_{4}\right) & \varepsilon_{H_{2}O}\left(\lambda_{4}\right) \end{array}\right]\;\left[\begin{array}{c} C_{\mathrm{Hb}O_{2}}\\ C_{\mathrm{HbR}}\\ C_{H_{2}O} \end{array}\right]\; & & \; \end{array}$$

We also assumed that biological tissues contained 70% water, reducing the above system to four equations with two unknowns. A pseudo-inversion of the equation with a least-square fit provided *C*, the hemoglobin concentration.

### Anatomical MRI

**Figure 3:**
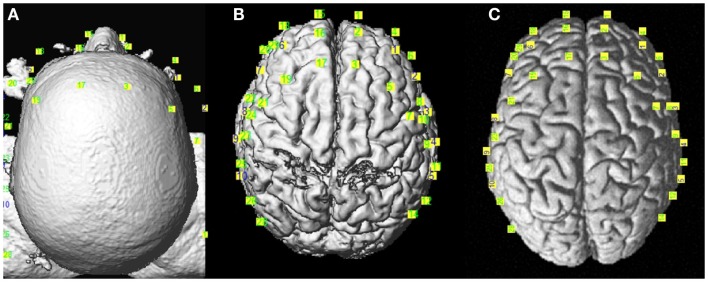
**Coregistration of the optodes’ positions from Brainsight©over the reconstructed anatomical images**. **(A)** Real, **(B)** MNI subject, and **(C)** MNI template spaces.

*Coregistration*: The anatomical images served two purposes in this study. First, at the individual level, we normalized the anatomical images to the MNI space including the subject’s fiducial coordinates. Then, to achieve better spatial resolution for fNIRS analysis, we projected the optodes’ positioning coordinates, collected from the Brainsight 3D camera, on the cortex (Figure 3). For group analysis, we needed to transform all images to the MNI template. These steps were performed using an in-house version of the algorithm of NIRS_SPM

### ASL data

The absolute CBF was measured in arbitrary units by the constant component of an ASL scan (ASL_0_) using the standard quantitative approach described by Wong et al. (52). This value was then converted to physiologically relevant units (mL blood/100 g tissue per minute) using a general kinetic model fit to the ASL signal (20, 49). This model defines a relationship between the measured signal and CBF_0_ assuming blood longitudinal relaxation time TI and fully relaxed magnetization M_0_ are known

CBF0=ASL06×106 ⋅ 2 ⋅ TI1 ⋅ exp (−T12T1b)⋅ M0b

where TI1 = 1400* *ms, TI2 = 2000 ms, and T1b = 1932 ms at 3 T and the factor 6 × 10^6^ converts the units for CBF_0_ to mL/min/100 g. The value for *M*_0b_, the fully relaxed blood magnetization, was calibrated using that of the white matter measured in the M_0_ scan (52):

$$M_{0b}=M_{0\mathrm{WM}}\cdot \frac{1}{\;\;\lambda \;\;}\cdot \exp\left(\mathrm{TE}\cdot \left(\frac{1}{T_{\mathrm{2WM}}}-\frac{1}{T_{\mathrm{2b}}}\right)\right)$$

with *M_0_*_WM_ the average value of the *M*_0_ scan in a region of interest (ROI) selected from the segmented white matter, the brain-blood partition coefficient for water λ = 0.9 mL/g (53), TE = 12 ms, *T*_2WM_ = 70 ms, and *T*_2b_ = 275 ms at 3 T. The blood flow measurement was then regressed against the functional NIRS data to evaluate its impact.

## Results

### Neuropsychological performance

There was no difference (*p* > 0.05) between the two age groups’ mean years of education (older = 16 ± 2.33 and younger = 16.95 ± 1.78). Both age groups were also matched for sex and consisted of 15 women and 8 men. They were compared on the neuropsychological measures of memory, vocabulary, and executive functions described earlier. The older adults performed worse on a number of subtests evaluating executive functions, but consistently with our hypothesis, the results of the vocabulary test showed no significant difference between the two groups (Table 1). Since responding correctly to the lexical-semantic decision task does not require planning or strategic changing skills, we expected these differences to have no impact on results.

**Table 1:** **Demographic variables and cognitive characteristics**.

	Variable	Young (*n* = 23) mean (SD)	Older (*n* = 23) mean (SD)	*F*-test (*p*-value)	*t*-test (*p*-value)
	MoCA	29.23(1.30)	27.27 (2.47)	0.0054	0.0037*
WAIS	Vocabulary	43.65 (7.68)	37.64 (14.75)	0.027	0.15
	Similarity	21.23 (5.08)	17.73 (3.92)	0.32	0.008*
	Block design	61.82 (3.15)	35.27 (7.93)	0.0002	0.000**
	Matrix	23.12 (2.87)	15.91 (5.70)	0.007	0.000**
	Digit spans	19.06 (4.34)	18.64 (3.99)	0.72	0.15
Hayling	Automatic	6.94 (0.24)	6.91 (0.29)	0.3	0.65
	Inhibition	5.47 (1.18)	4.82 (1.65)	0.1	0.15
	Final score	19.29 (1.61)	17.5 (2.70)	0.47	0.12

Results from neuropsychological batteries by age group. MoCA, Montreal Cognitive Assessment; WAIS, Wechsler Adult Intelligent Scale; Hayling, test for inhibitory control. *p < 0.05, **p < 0.0001.

### Task performance

**Figure 4:**
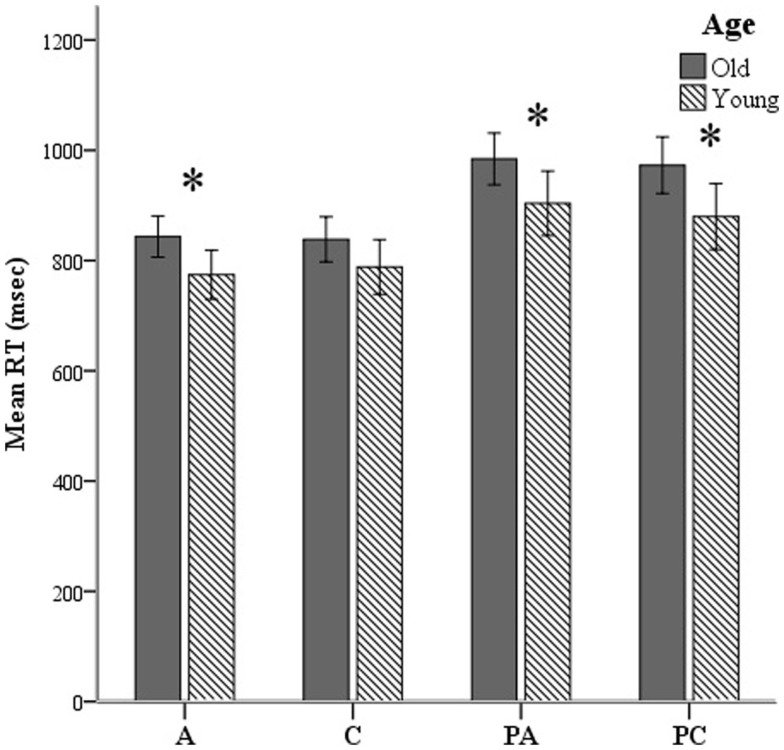
**Mean reaction times (RTs) shown here over all lexical-semantic conditions of the task: A, abstract; C, concrete; PA, pseudo-abstract (pseudo-words derived from abstract stimuli); and PC, pseudo-concrete (pseudo-words derived from concrete stimuli)**. *p < 0.05.

Both groups performed equally accurately across conditions except on pseudo-words derived from concrete words [F(1, 55) = 6.1, *p* = 0.017]. Young participants were faster in all conditions except for concrete words. We applied a two-standard-deviation cutoff on the RTs of correct responses. RTs for correct trials are presented in Figure 4. Results from two 2-way ANOVAs showed no age × lexicality [F(1, 176) = 0.621, *p* > 0.05] or age × condition [F(1, 176) = 0.275, *p* > 0.05] interactions. There was a simple effect of lexicality [F(1, 179) = 11.4, *p* = 0.001], irrespective of age.

### Time-resolved spectroscopy

We calculated the absolute oxy- and deoxyhemoglobin concentrations ([HbO_2_] and [HbR], respectively) as well as oxygen saturation (SatO_2_) in both right and left prefrontal lobes covered by our four-channel TRS patch. Considerable intersubject variability was observed in the measures of hemoglobin concentrations, as previously reported in the literature (34). Group mean comparisons were inspected for the homogeneity of variance assumption. When the variance test of homogeneity was significant, the *p*-value of the unequal variance for one-tailed *t*-tests was reported. TRS measures of right and left frontal areas were analyzed separately. Older adults showed a resting [HbO_2_] and [HbT] (HbT = HbO_2_ + HbR) decrease in the left hemisphere compared to the right and an overall reduced [HbO_2_] compared to young adults (Table 2). A one-tailed comparison revealed a decrease in SatO_2_ in both left and right prefrontal lobes for the elderly group (*p* = 0.045 and 0.029, respectively). Because we observed a different trend in hemispheric changes in the measured hemoglobin concentrations across age, we applied a two-way ANOVA to investigate the effect of the baseline physiology of aging and laterality. The results showed no significant interaction between age and laterality.

**Table 2:** **Results from TRS measurements**.

	LH		RH	
	Old	Young	Old	Young
[HbO2]	40.9 (±1.7)	49.1 (±2.1)	42.6 (±1.8)	46.5 (±1.3)
*t*-Test	0.002**		0.039*	
[HbR]	25.8 (±1.1)	28.2 (1.0)	25.9 (±1.1)	25.6 (±1.0)
*t*-Test	0.056		0.428	
SatO2	61.2 (±0.7)	63.3 (±0.9)	62.2 (±0.7)	64.6 (±1.0)
*t*-Test	0.045*		0.029*	

Oxy- and deoxyhemoglobin concentrations and oxygen saturation calculated in both hemispheres from TRS measures in micromoles, *p < 0.05, **p < 0.001.

### Resting-state CBF

**Figure 5:**
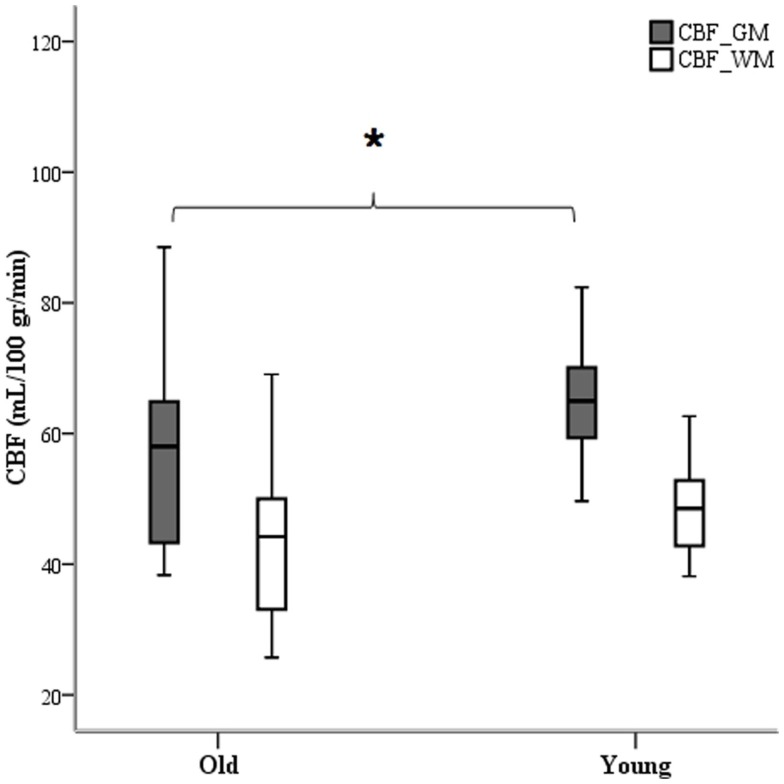
**White and gray matter segmented images as ROI masks were used to calculate mean CBF**. Only gray matter blood perfusion yielded a difference between age group averages. *p < 0.05.

Analysis of tagged ASL images revealed different group averages over the segmented white and gray matter anatomical images applied as explicit masks. Mean baseline CBF calibrated with the use of individual voxel values of the *M*_0_ sequence was computed for both age groups and a significant difference was observed for global (*p* = 0.001) and gray matter (*p* = 0.02) blood perfusion (Figure 5). To investigate regional effects, we calculated mean CBF within regions of interest (ROIs) defined by the optical helmet. Temporal and frontal ROIs were created by applying a 35 mm diameter disk around optical channels covering the temporal and frontal regions. At rest, older adults had significantly lower blood flow (*p* = 0.01) in both temporal and frontal lobes than their younger counterparts.

### Functional optical recordings

Stimulus-dependent activation of all 58 channels was measured within the areas covered by the optodes. Significant changes were defined at *p*-values of less than 0.05. The activation map was then obtained via interpolation of the beta values calculated from the GLM model over the localized optodes coregistered on the MNI template, although a lack of spatial resolution made it difficult to identify activation areas with any precision.

The regions of activation for group average, obtained from the intersection of individuals’ activation maps, were impacted by variability induced in the positioning of optodes. Thus, a smaller group-level significant activation map was obtained, although the pattern of activation followed the same language network areas at the individual level. Brain regions activated by the lexical-semantic task were partially different for word and pseudo-word stimuli. In response to semantic word processing, a decrease in [HbR] concentration was observed in the elderly adults group in the left frontal region, at the intersection of the right inferior frontal gyrus (IFG) and the superior temporal gyrus (STG). In young adults, pseudo-word stimuli generated diminished [HbR] and increased [HbO_2_] in the left IFG but an inverse response (decreased [HbO_2_] and increased [HbR]) in the left inferior temporal (IT) and frontal lobes. In contrast to elderly participants, activated areas remained in the temporal sulci for other types of stimuli (concrete and abstract) but again in an inverse fashion.

An ANOVA for the main effect of age depicted significant [HbO_2_] and [HbR] changes in both right and left hemispheres when older and younger adults were compared. Activation differences were mainly found in the bilateral dorsolateral prefrontal cortex (DLPFC) and IFG, and right posterior middle temporal and occipitotemporal gyri. Younger participants showed increased [HbR] and decreased [HbO_2_] in the DLPFC and IFG in response to the semantic processing task (so-called inverse response). Conversely, pseudo-words led to a significant right ventral anterior premotor cortex decrease [HbR], which could be interpreted as an effort to analyze the stimulus by covert reading.

#### TRS regressors

**Figure 6:**
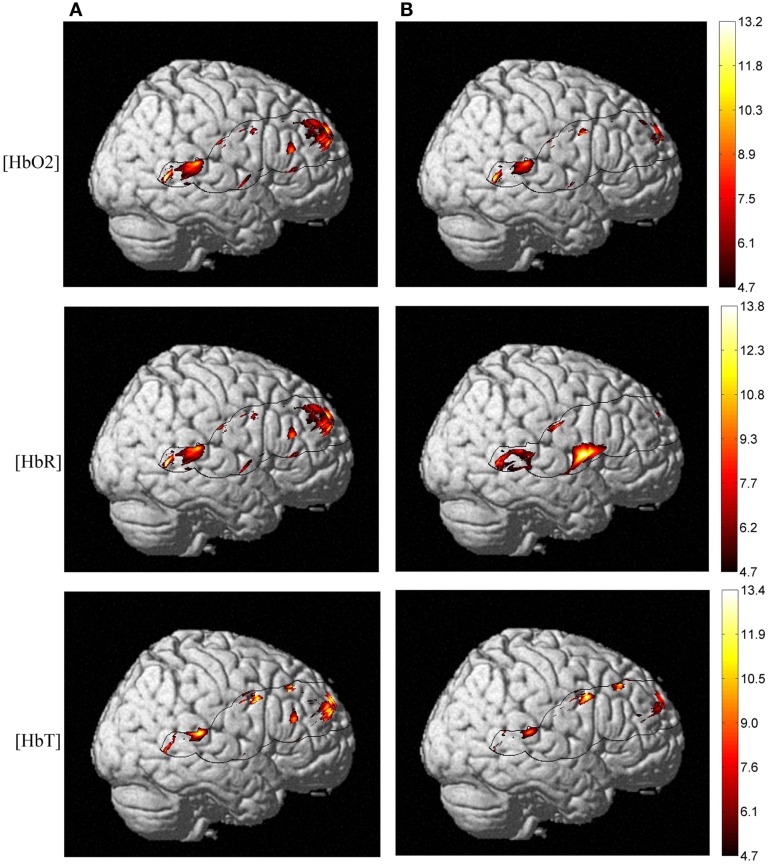
**NIRS activation maps of the hemoglobin concentrations for the main effect of age**. With TRS measures of baseline hemoglobin concentrations regressed against stimulus-dependent activation, we observed a different pattern of posterior-inferior alteration as an effect of age. But this pattern is more pronounced at the [HbO_2_] and [HbR] level and not for [HbT], which is an estimate of the cerebral blood volume. Panel **(A)** shows Δ[Hb] age differences without taking into account baseline physiology measures and panel **(B)** depicts activation differences once applying TRS regressors into ANOVA.

**Figure 7:**
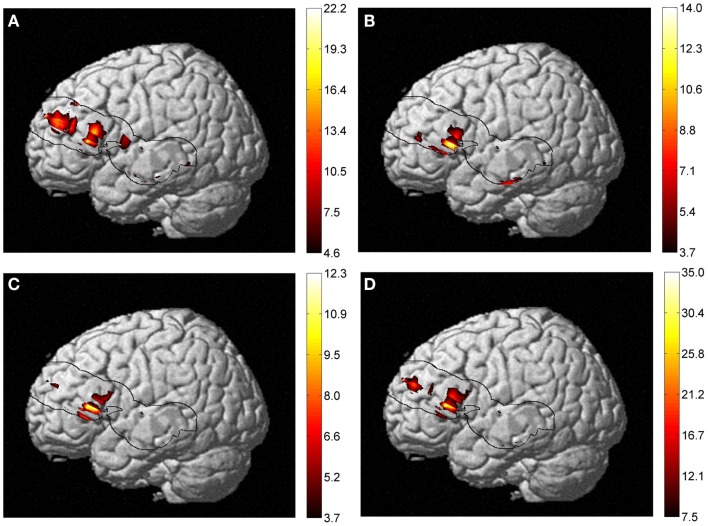
**Results of a two-way ANOVA on [HbR] examining the factors of age and condition (of lexical-semantic task) on the activation maps**. **(A)** Main effect of age, **(B)** main effect of condition, **(C)** interaction between age and condition, and **(D)** effect of age on pseudo-word processing are shown. An IFG age difference was present in all four types of analysis but with different extents of activation. A *post hoc* analysis revealed an effect of age on pseudo-word processing in the DLPFC and IFG. No significant [HbO_2_] or [HbT] differences were observed.

Taking into account the measures of baseline physiology from the TRS system by including individual measures as regressors, we investigated their contribution to the observed age difference in [HbO_2_] and [HbR] stimulus-dependent changes. In the right hemisphere, the main effect of age faded significantly in the frontal lobe for both [HbO_2_] and [HbR], and [HbR] seemed to become more significant at the intersection of the IFG, STG, and caudal border of the anterior central gyrus (BA43) (Figure 6). In the same fashion, a *post hoc* ANOVA on the effect of age on lexicality (pseudo-words), revealed significant [HbR] differences in right BA43. However, there was no such effect on total hemoglobin concentration [HbT] differences with regressors, which could suggest that the blood supply alters with age, if we use [HbT] as an estimate of cerebral blood volume. In the left hemisphere, there was an age difference in the frontal lobe (Figure 7), with no effect of TRS regressors. An interaction between age and condition was present only at the [HbR] level, with changes found in the IFG. A *post hoc* analysis revealed an effect of age on pseudo-word processing in the DLPFC and IFG. In the left hemisphere, we did not find any significant differences in the temporal regions.

### Correlation analyses

A correlation coefficient was calculated to examine the associations between age, performance and baseline physiology (ASL and TRS measures). Here, we sought relationships between RT and physiological measures while controlling for the effects of age and performance. The partial correlation coefficient between RT and [HbO_2_], [HbR], SatO_2_, and CBF was calculated by adjusting for age and performance scores. We presumed that both variables of correlation were linearly related to age and performance on the neuropsychological tests.

**Figure 8:**
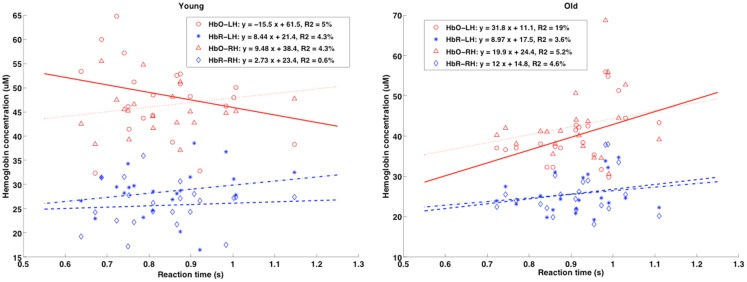
**Scatterplots of mean RT (*x*-axis, in seconds) vs. TRS baseline physiology measures (absolute [HbR] and [HbO]; *y*-axis, in micromolar) for the younger and elderly groups are shown for both right and left hemispheres (RH and LH)**. Pearson’s *r* coefficients showed no significant correlation between baseline [Hb] and RT, but different trends were present when comparing age groups.

Inspection of the correlations between RT and CBF measures revealed no significant relationship. Pearson’s coefficient of correlation between RT and [HbO_2_], [HbR] and SatO_2_ dropped significantly once we removed the effect of age (from *r* (42) = 0.35 to *r* (40) = 0.06). It is interesting to note that, even though there were no significant correlations between the variables under investigation, the variations tended to move in opposite directions when younger and older adults were compared (see Figure 8). The tendency graphs revealed that slower responding elderly participants had slightly elevated baseline [HbO_2_], [HbR], and SatO_2_, whereas younger individuals showed the opposite pattern (i.e., slower respondents had lower baseline levels).

## Discussion

The goal of this study was to evaluate the validity of the assumption that, when different age groups are compared, the hemodynamic response is a direct indicator of neuronal activity in response to a cognitive stimulus. The main result showed that, when each participant’s baseline physiology is taken into account, the degree and extent of neural activity varied significantly in the right hemisphere – an observation that could change the interpretation of less asymmetrical language-related neural engagement. The present study supports the reliability of single-word processing studies using fNIRS while urging caution in the interpretation of functional signals. RTs and accuracy on the lexical-semantic task showed the presence of a lexicality effect for both age groups. However, a different pattern of brain activation was found for young and older participants. We observed that searching for semantic knowledge while processing words vs. pseudo-words in the lexical-semantic task engaged largely overlapping brain regions in the left hemisphere, including the posterior temporal gyri, which is consistent with the notion that activity in the left temporal regions is linked to the verbal abilities of word retrieval rather than the lexical class to which the stimulus belongs (54).

A right frontal [HbT] difference between the young and older samples could be in line with findings from TRS measures for lower oxygen saturation in the elderly group. In response to neural activity and to compensate for reduced baseline HbO_2_ concentrations and CBF, older adults may need greater blood volume in their compensatory networks. Thus, controlling for baseline physiology is expected to make the interpretation of results more reliable.

The presence of right DLPFC, frontotemporal cortex, occipitotemporal and angular gyri [homolog to areas known as the visual word form area (55)]; activation in the elderly cohort is compatible with the idea that bilateral neural activity increases with age (56, 57), although it is important to note that this age-related pattern of activity was observed by means of Δ[HbR] differences in the frontotemporal cortex and [HbO_2_] variation in the DLPFC. Without [HbT] variation and Δ[HbR] differences, it could be assumed that this latter observation is due to mere neural activity and not to baseline physiological differences.

In the contrast between pseudo-words and real words, we found an age-different cluster of activation in the right IFG–STG intersection, bilateral DLPFC, and left IFG, which is consistent with previous findings claiming for the compensatory mechanism of the brain activity with effort in accordance with the behavioral finding that RTs are longer for pseudo-words (56, 58). It can be noted that the hemispheric laterality for language is relative and that it relies on a stronger engagement of the left hemisphere for syntactic and semantic processing, which nevertheless coexists with right-hemisphere activation. Thus, when the different age groups are compared, the difference in hemispheric patterns of activation could express over-recruitment of reserve networks, meaning that activation becomes less lateralized. Moreover, controlling for the baseline physiology strengthened the analyses, as it revealed differences in mere neural activity triggered by the task and not in the absolute hemoglobin concentration differences (data shown in TRS results). However, a question remains about whether bilateral frontal activity in older adults is driven by task difficulty *per se* or whether baseline physiological differences compared to their younger counterparts led to this differentiation.

The inverted hemodynamic response in the young group could be due to local coarse regulation of oxidative metabolism, provoked by the increase in neuronal activity, which is overwhelmed by an increase in CBF. In this regard, Woolsey and colleagues (59) postulated that there is a hemodynamic “steal” effect. The “steal phenomenon” may explain this observation by accounting for subsequent CBF changes: some arterioles were metabolically dilated while others in neighboring areas were constricted (60, 61).

More generally speaking, this study confirms the usefulness and sensitivity of hemodynamic-based corrected fNIRS imaging in investigating the neurofunctional reorganization of word processing and cognition with age. The same technique could be used after brain lesion and in recovery. This study also confirms the existence of a form of neurofunctional reorganization that corresponds to compensatory mechanisms, allowing for the preservation of linguistic abilities despite the neurophysiological changes present in aging.

In summary, while the combination of absolute and relative changes in hemoglobin concentration eliminates some of the assumptions previously required in NIRS data analysis, further improvements are needed. Future studies will be augmented by parallel ASL measurements of blood flow variation during brain activation, to provide a detailed measurement of quantitative events accompanying neuronal activation. Individual brain volume analyses would also provide a better estimation of the optimal source-detector distances for different age groups. In the case of atrophy in the elderly population, it is important to correct for the source-detector distances to allow for optimum light penetration and differences in light scattering and absorption.

## Conflict of Interest Statement

The authors declare that the research was conducted in the absence of any commercial or financial relationships that could be construed as a potential conflict of interest.
